# A Rare Case of Chondroma of the Cartilaginous Nasal Septum

**DOI:** 10.7759/cureus.70830

**Published:** 2024-10-04

**Authors:** Sakthimurugan Sankar, Shyam Sudhakar Sudarsan, Dinesh Ram R

**Affiliations:** 1 Otorhinolaryngology, Saveetha Institute of Medical and Technical Sciences, Chennai, IND

**Keywords:** cartilage, chondroma, head and neck neoplasms, nasal septum, nose

## Abstract

Nasal chondromas, benign and slow-growing tumors, have been scarcely documented in medical literature, with fewer than 150 cases reported. This case study presents a rare instance of nasal septum chondroma arising from the anterior part of the septum. A 50-year-old woman presented with complaints of bilateral nasal blockage for five months, with no history of trauma or nasal pain. Imaging and histopathological evaluations confirmed the diagnosis of a benign chondroma. The tumor was successfully removed through a sublabial approach to the pyriform aperture, preserving the nasal framework, and the patient remained asymptomatic for 18 months postoperatively with no recurrence. While nasal chondromas are benign, they have the potential for local invasion and sarcomatous transformation, necessitating long-term follow-up. This case underscores the importance of thorough histopathological examination and careful surgical intervention to ensure a favorable prognosis.

## Introduction

Cartilaginous tumors commonly appear in the long bones, pelvis, sternum, ribs, and scapula. However, around 10% of cases occur in the head and neck region, with the larynx and ethmoidal sphenoidal region being the most prevalent sites [1]. Within this category, nasal chondroma, a notably rare and benign tumor, is primarily identified in the nasal septum. The inaugural case documentation dates back to 1842 by Morgan, and since then, only about 150 cases have been reported in the medical literature [1]. Cartilaginous tumors of the nasal septum are highly uncommon and typically originate from the posterior region. Here, we present a case of chondroma arising from the anterior part of the septum, an exceedingly rare scenario. The tumor exhibited well-defined, homogeneous characteristics and was extensively excised using a sublabial approach. Despite the rarity of such tumors, a traumatic etiology, although least accepted, appears to be the most appropriate for oncogenesis in this case. We advocate for a comprehensive histopathological examination of the tumor to detect any early sarcomatous changes.

## Case presentation

**Figure 1:**
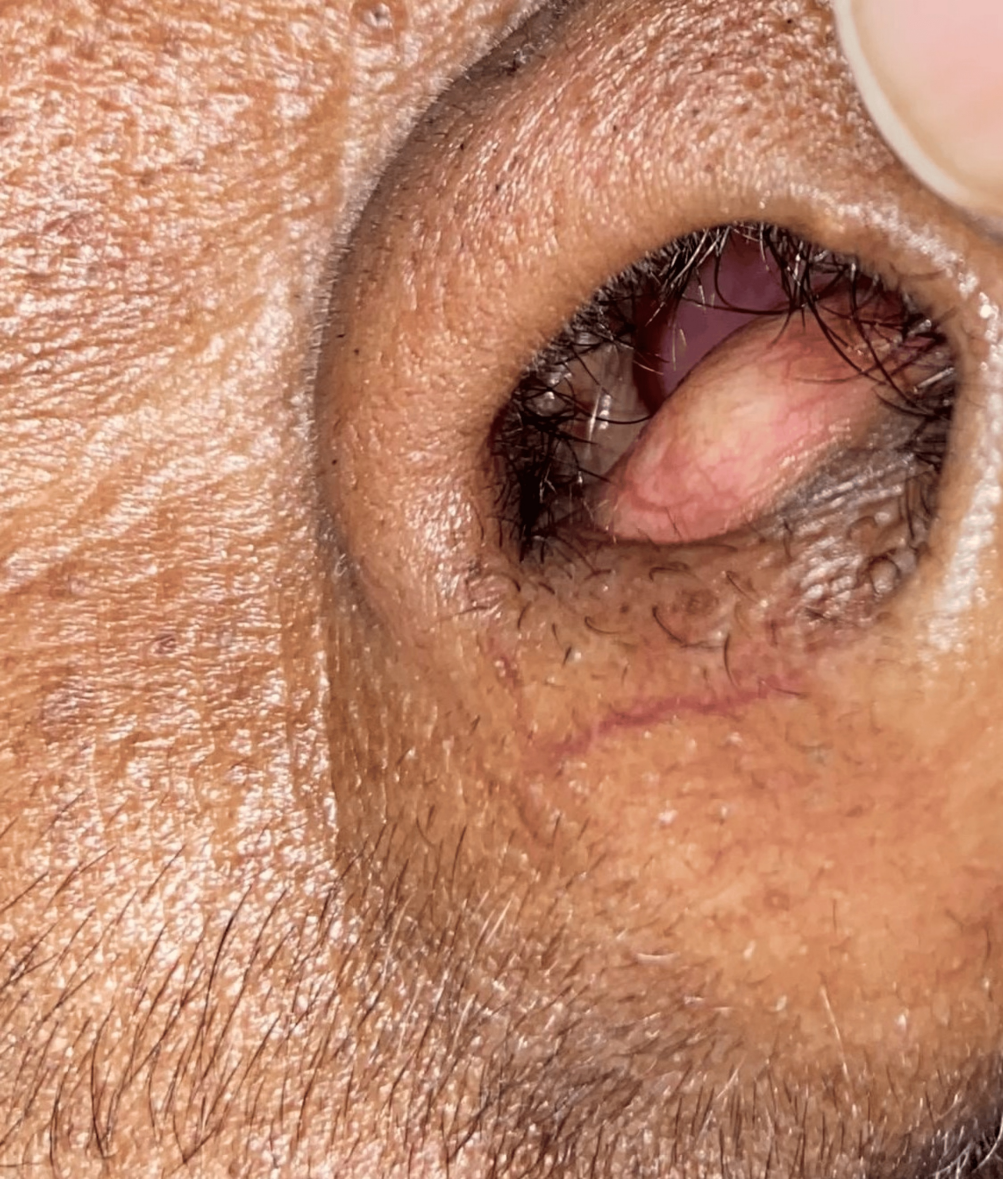
Preoperative image of the nasal cavity showing swelling in the anterior nasal septum - right side

**Figure 2:**
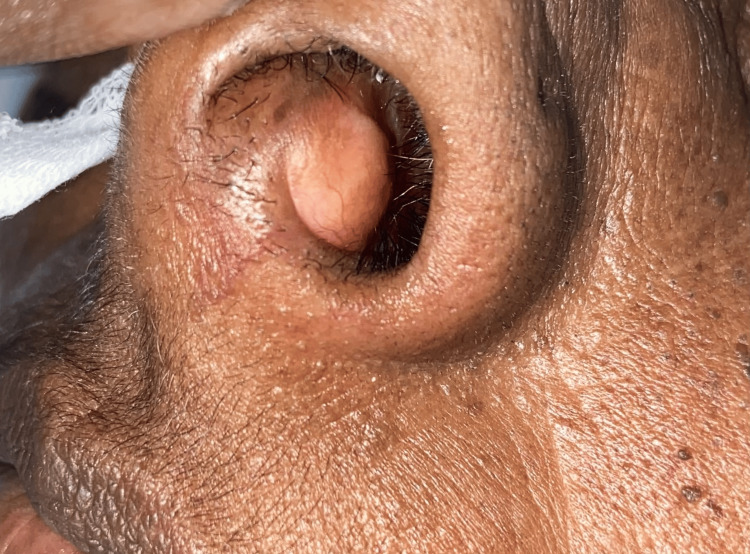
Preoperative image of the nasal cavity showing swelling in the anterior nasal septum - left side

A 50-year-old female presented to our hospital with complaints of bilateral nasal block for the past five months. She did not experience nosebleeds, runny nose, nasal pain, any history of trauma, eye-related symptoms, or facial deformities. Local examination and nasal endoscopic examination revealed a well-defined, smooth, firm, pale mass arising from the midline of the anteroinferior part of the nasal septum (Figures 1, 2).

On probing, it was sensitive and did not bleed. It did not have any contact with the turbinate and sinus. A neck examination showed no cervical lymphadenopathy.

**Figure 3:**
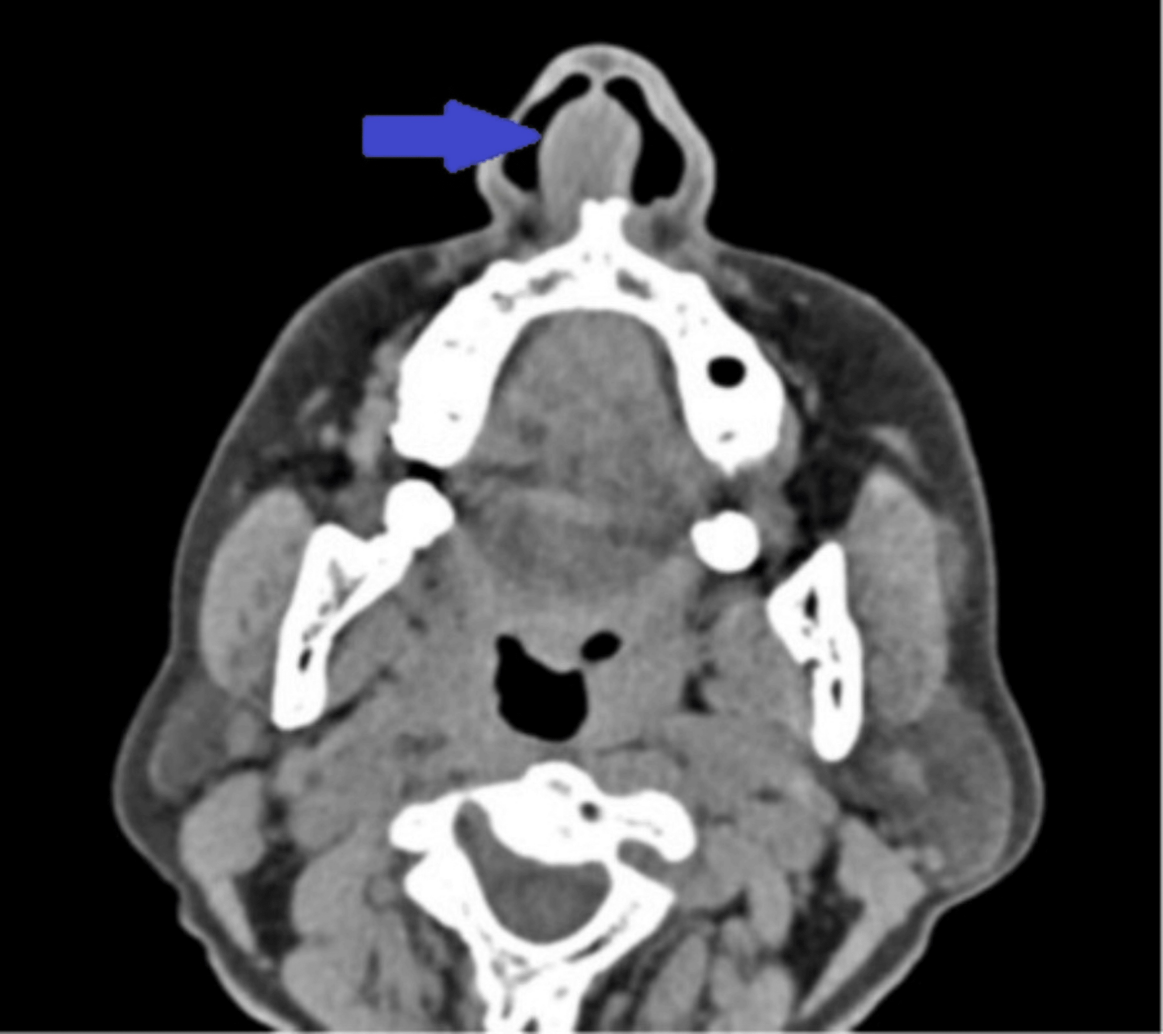
Computed tomography image of the nose and paranasal sinuses showing a solid dense swelling in the septal cartilage

Computed tomography of the nose with paranasal sinuses was done, which showed a solid dense swelling noted in the midline with the epicenter in the anteroinferior aspect of the septal cartilage, which was reported as a benign nasal septal swelling with features suggestive of nasal septal chondroma (Figure 3).

**Figure 4:**
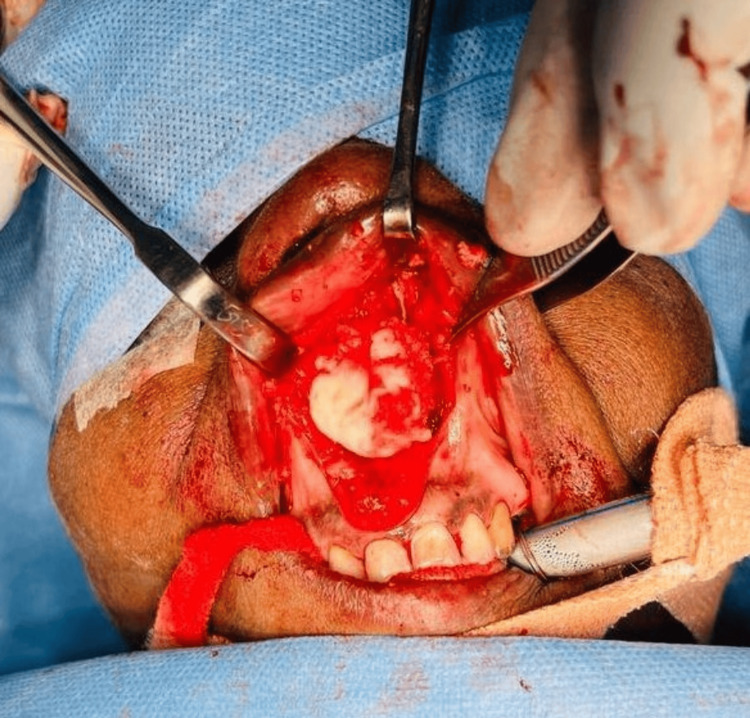
Intraop image showing septal swelling through the sublabial incision

Excision of the mass in-toto through a sublabial incision and approach to the pyriform aperture (Figure 4) under general anesthesia was done and the mass was removed in its entirety.

**Figure 5:**
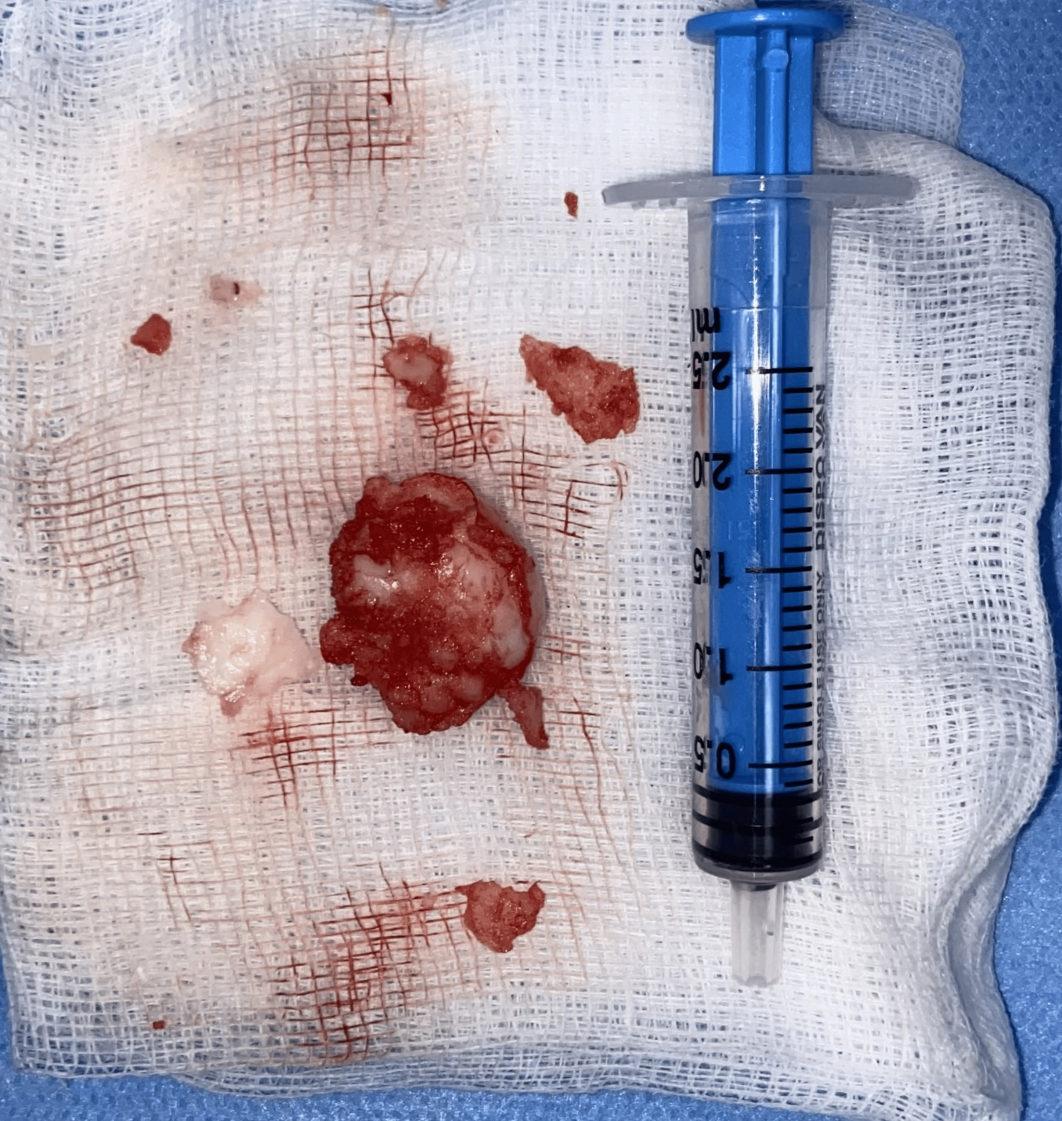
Gross image of the excised specimen of the chondroma

The perichondrium was also removed, but the healthy cartilage was preserved with proper inferior and anterior struts to preserve the septal framework and prevent columella or tip deformity since no bone or cartilage destruction was noted. A tiny rent near the anterior nasal spine area was noted on the nasal side, which was due to submucosal dissection; this was closed using 4-0 vicryl. A large, gray-white soft tissue fragment with approximate dimensions of 2.5x2x1 cm and a few smaller fragments measuring between 0.5 to 1 cm (Figure 5) were sent for histopathological examination. The sublabial wound was closed in layers.

**Figure 6:**
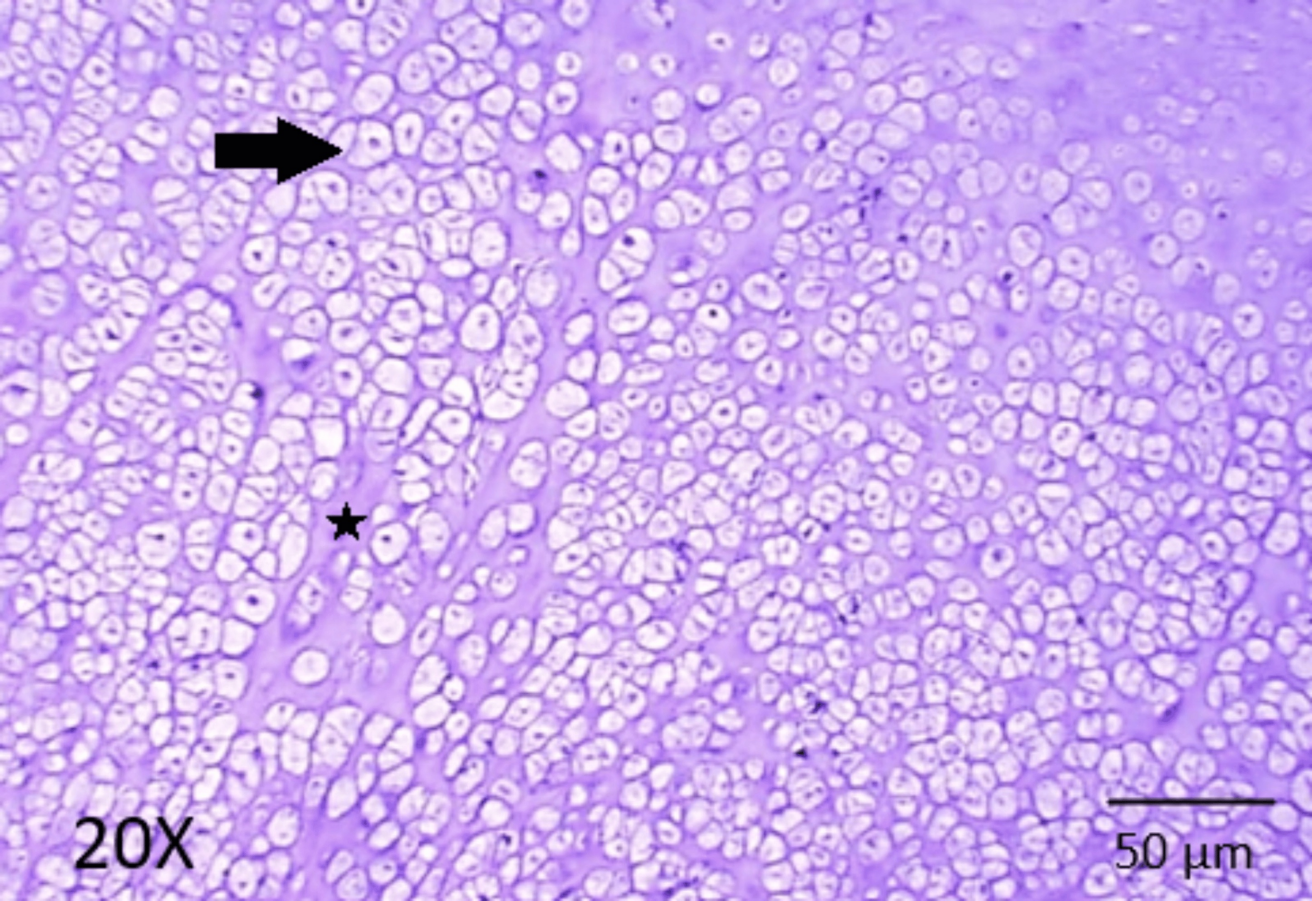
Histopathological section showing a fairly circumscribed neoplasm composed of chondrocytes in lobules separated by a cartilaginous matrix No evidence of mitosis or necrosis is noted. The arrow points at the chondrocyte cell and the asterisk shows the cartilaginous matrix. Staining with hematoxylin and eosin at 20x magnification

The postoperative period was uneventful. On recovery and follow-up, no perforation or fistula was noted at the surgical site. The histopathological analysis of the specimen revealed nodules of mature cartilage tissue with lacunae containing benign chondrocytes (Figure 6), along with fibrous collagenous tissue and blood vessels.

**Figure 7:**
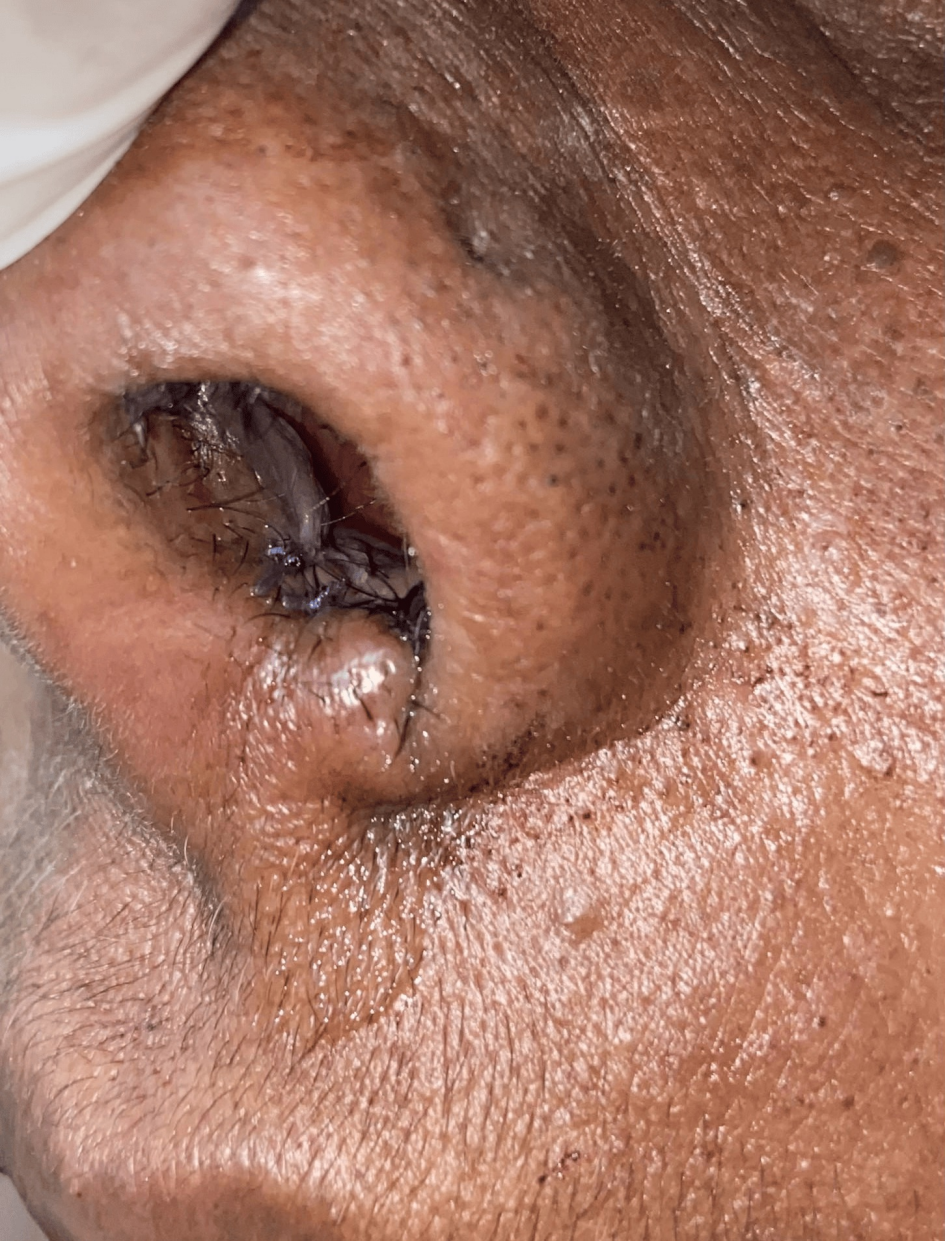
Postoperative image showing the left nasal cavity

**Figure 8:**
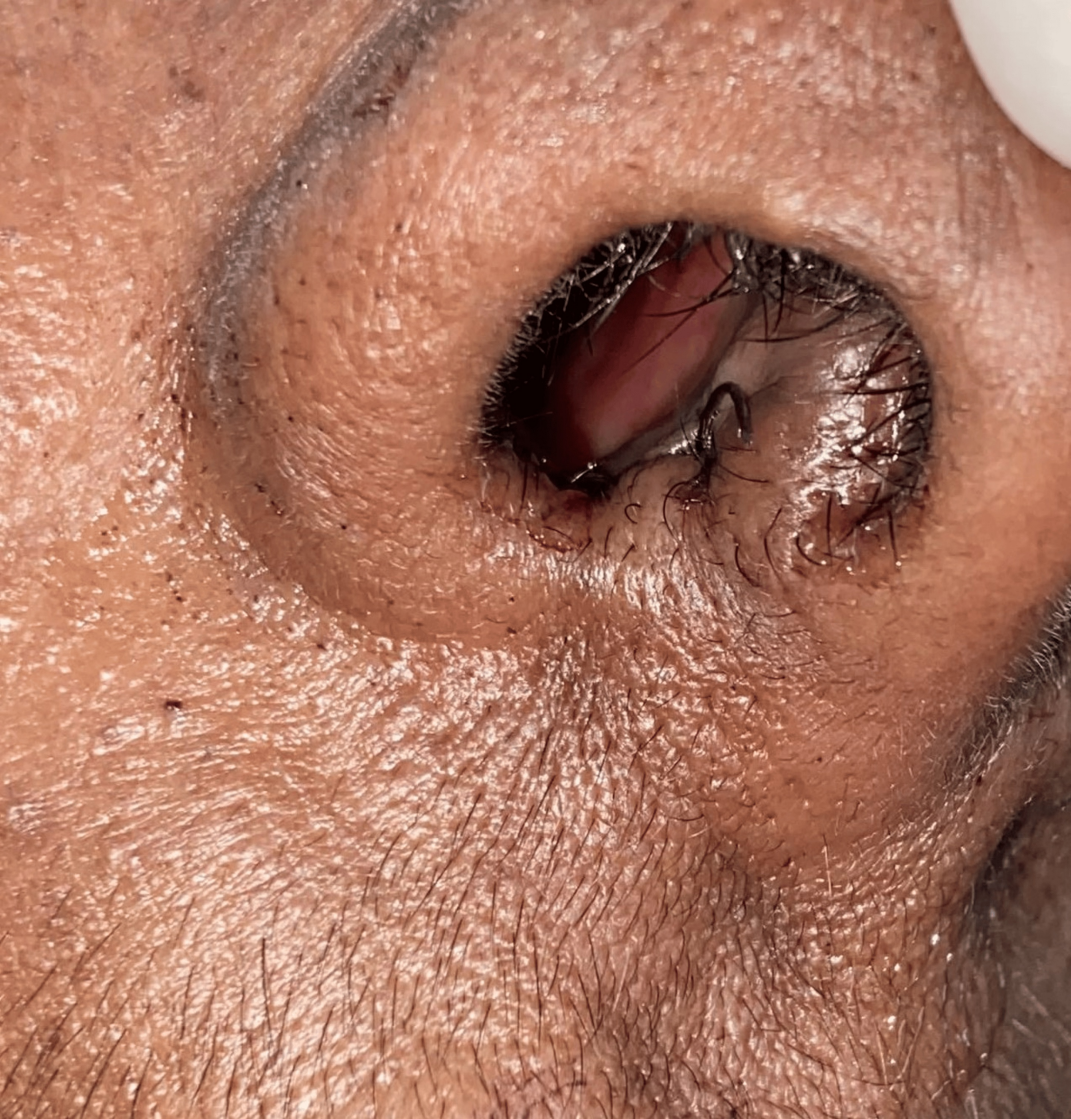
Postoperative image showing the right nasal cavity

These features were indicative of a chondroma. The postoperative images are shown in Figures 7, 8. The patient has been regularly monitored and has remained symptom-free for the past 18 months, with no signs of recurrence during follow-up.

## Discussion

The differential diagnosis for a mass in the nasal cavity encompasses both inflammatory and neoplastic conditions. Soft-tissue masses in the nasal cavity, with or without bone destruction, can result from nasal polyps, fungal infections, rhinosporidiosis, tuberculosis, Wegener's granulomatosis, and lethal midline granuloma [2]. Cartilaginous tumors in the head and neck region most commonly affect the ethmoid sinus (50%), followed by the maxilla (18%), nasal septum (17%), hard palate and nasopharynx (including the sphenoid and eustachian tube; 6% each), and alar cartilage (3%) [3].

The location, size, and growth rate of the tumor all affect nasal chondroma symptoms. Slow-growing chondromas of the nasal cavity are characterized by symptoms such as epistaxis and nasal blockage. Extension into the orbits can result in blindness, diplopia, proptosis, and epiphora. Patients with maxillary involvement may experience toothaches and poorly fitting dentures. Imaging is essential for evaluating both the bone and soft-tissue characteristics of the tumor and determining its full extent. A contrast-enhanced CT scan of the nose and paranasal sinuses is useful for assessing the tumor’s spread. Nasal chondromas generally do not show significant radiopacity. These tumors typically appear well-circumscribed and appear fairly homogenous on computerized tomography scans [4]. The diagnosis of a nasal chondroma relies on a combination of clinical, radiologic, and pathologic findings. In cases where the diagnosis is uncertain, MRI can be useful, as chondromas often show increased signal intensity on T2-weighted images [5]. Macroscopically, chondromas are smooth, firm, and lobulated, with a texture described as "gritty" or similar to a ripe pear. Microscopically, the cartilage cells are small, with pale, vacuolated cytoplasm and small, dark-stained nuclei. A process of amitotic division may be evident in some fields where cartilage cells are binucleate, although the majority are monocellular and mononucleate.

The exact pathophysiology of chondroma is not known. The prevailing theory for the origin of chondromas is that they develop from residual embryonic cartilage cells that persist and do not undergo resorption during endochondral ossification [6]. There have been reports of hormonal influences, such as those associated with pregnancy and menopause [7], affecting the growth of chondromas in intracranial tumors, including those at the skull base. However, nasal septum chondromas presenting during menopause have not been documented previously. The enlargement of these tumors during pregnancy may be attributed to physiological changes that increase blood vessel engorgement, potentially leading to tumor growth. Additionally, fluctuations in hormone levels, including estrogen and progesterone, might impact tumor development. Plain X-rays and CT scans are useful for evaluating the tumor’s extent, but a biopsy remains the definitive method for diagnosis. Although chondromas are typically benign, they differ from other benign tumors in that they can be locally invasive, tend to recur after removal, and may exhibit sarcomatous transformation. In cartilaginous tumors of the nasal passages and other skeletal locations, less than 5% of tumors that appear clinically and histologically benign ultimately progress to malignancy.

For large nasal chondromas, lateral rhinotomy is generally the preferred surgical approach. In contrast, smaller lesions can be safely and effectively managed with an endoscopic approach [8]. Research shows that with proper treatment, the prognosis for nasal chondromas is generally positive, and recurrences are rare [3]. In this case, complete surgical excision was performed, resulting in a favorable outcome.

## Conclusions

The standard of care for cartilaginous tumors, in our case, is complete surgical excision. Cartilaginous tumors are generally radioresistant. In most cases, radiotherapy is utilized when a patient's illness cannot be cured surgically. As chondromas have a predisposition for sarcomatous alteration, long-term follow-up is strongly encouraged. Although metastasis is uncommon and happens later in chondrosarcoma, it usually affects the lungs. Both benign and malignant types of the disease spread locally. To find any early sarcomatous alterations, a thorough histological investigation of the tumor is recommended. Either lung problems or intracranial spread of the tumor causes death. With proper and complete surgical excision, the prognosis is good and recurrence is rare.
